# Interpretable machine learning for self-reported reduced uncorrected visual acuity in school students

**DOI:** 10.3389/fpubh.2026.1872000

**Published:** 2026-10-26

**Authors:** Yangzhou Li, Lin Zhang, Jikai Wang, Shihao Fu, Yalan Liu, Lingyan Yuan, Mengliang Ye

**Affiliations:** 1 Chongqing Medical University, Chongqing, China; 2 Nan'an District Center for Disease Control and Prevention, Chongqing, China

**Keywords:** internal–external cross-validation, interpretable machine learning, probability calibration, public health, questionnaire, school eye health, self-reported visual acuity

## Abstract

**Introduction:**

Myopia and reduced vision among children and adolescents place a substantial burden on public health, particularly where eye-care resources are limited. We evaluated an interpretable questionnaire-based framework for modelling self-reported reduced uncorrected visual acuity.

**Methods:**

The analysis included the first eligible record for each of 7,118 students from seven schools across three survey rounds. Sixteen demographic, familial and behavioural predictors were used to model an affirmative response to a question about uncorrected visual acuity below 5.0 in at least one eye. Internal-external cross-validation (IECV) comprised seven outer folds, each holding out one school. Preprocessing, model selection, probability calibration and Youden threshold estimation were confined to the training schools.

**Results:**

The selected workflow achieved an area under the receiver operating characteristic curve (AUC) of 0.706 [95% confidence interval (CI) 0.694-0.718], a Brier score of 0.212, sensitivity of 0.730 and specificity of 0.575. The academic-year-only benchmark achieved an AUC of 0.611 and a Brier score of 0.233. Stage-specific AUCs ranged from 0.633 to 0.680, with substantial variation in sensitivity and specificity. Applying the same development procedure to the full cohort selected CatBoost. SHapley Additive exPlanations (SHAP) identified academic year, parental myopia and desk-chair adjustment as leading contributors to its predictions.

**Discussion:**

Questionnaire responses provided predictive information about self-reported visual status beyond academic year, with moderate overall discrimination and variation across educational stages. These findings provide a basis for further evaluation of the framework in school eye-health research.

## Introduction

School-based surveys in China have documented a substantial burden of myopia among children and adolescents (1). Global projections indicate that this burden will continue to increase, reinforcing the need for accessible prevention and eye-health services (2). International consensus emphasises coordinated prevention, early detection and public education (3). Evidence of myopic maculopathy progression in children with high myopia highlights the consequences of severe disease (4), while a large school-based survey in Tianjin illustrates the population-level burden in China (5). Access to follow-up eye examinations and refractive care may be constrained by the availability of specialists and equipment. These constraints motivate efforts to make better use of routinely collected information and strengthen coordination between schools, families and eye-care services.

School eye-health programmes require practical approaches that integrate prevention, assessment and health communication. Economic modelling of increased outdoor activity highlights the importance of evaluating preventive strategies in resource-constrained settings (6). Research on digital phenotyping has explored potential clinical applications (7), including app recommendations and sustained engagement (8). In eye health, the World Health Organization (WHO) MyopiaEd programme illustrates the use of digital communication with parents (9). Studies of outdoor activity (10), school environments (11) and screen exposure (12) provide a basis for examining behavioural and environmental information. In the present study, behavioural phenotyping refers specifically to the analysis of structured questionnaire responses. An interpretable modelling approach can show how these recorded characteristics contribute to predicted visual status.

We developed an interpretable modelling framework using readily obtainable demographic, familial and behavioural information. We examined its ability to distinguish students by self-reported visual status, its predictive contribution beyond academic year, and variation in performance across schools and educational stages. Gradient boosting machines (GBM), CatBoost and logistic regression were compared through nested school-grouped resampling within internal–external cross-validation (IECV). SHapley Additive exPlanations (SHAP) was used to explain predictions from the final model at both global and individual levels. By focusing on visual status reported at the time of the survey, this study characterises the information available from routine school eye-health questionnaires.

## Methods

### Study design and participants

This multicentre cross-sectional study analysed questionnaire records from school-health monitoring in Nan’an District, Chongqing, China. The 2021, 2022 and 2023 monitoring archives corresponded to surveys conducted in October–November 2021, February–March 2023 and October–November 2023, respectively. Eligibility was defined by the questionnaire’s target population: students in primary grades 4–6 and the recorded junior-high, senior-high and vocational grades. School names and annual rosters were linked to establish consistent school membership and identify students across survey rounds. Retaining each identifiable student’s first eligible record yielded 7,118 students from seven schools. The study was conducted in accordance with the Declaration of Helsinki and approved by the Medical Ethics Committee of Nan’an District Center for Disease Control and Prevention. Written informed consent was obtained from adult participants and from the parents or legal guardians of participants younger than 18 years.

### Questionnaire-based behavioural phenotyping

The structured questionnaire covered demographic characteristics, the school environment, academic activities, reading posture, screen use, lighting, lifestyle, family history and schoolbag use. A fixed set of 16 predictors was used for this analysis; their definitions are provided in Supplementary Table S1. Academic year, total daily sleep duration and total daily indoor-sitting duration were treated as continuous variables. The remaining 13 predictors were represented by separate indicators for their response categories. Academic year was coded as 4–6 for eligible primary-school students, 7–9 for junior-high students and 10–12 for senior-high and vocational students. School and individual identifiers were used solely to construct the analytical sample and define resampling groups. Sex and location during school breaks were included in descriptive analyses but were not model predictors.

### Data quality control

Data quality checks covered outcome values, categorical response ranges, school membership, repeated participation and the components of reported daily durations. In the primary analytical cohort, the raw fields for all 16 predictors and the outcome contained no blank entries, and all categorical responses were within the documented questionnaire options. Descriptive statistics for sex were based on questionnaire responses; five responses differed from the linked roster. Sex was not included in the prediction models.

The questionnaire recorded duration in hours and minutes separately. Total duration in hours was calculated as the hour component plus the minute component divided by 60. Valid responses required an hour component of 0–24, a minute component of 0–59 and a total duration of no more than 24 h. Derived durations that failed these checks were treated as missing, while the original responses were retained unchanged. This affected five sleep-duration values and 72 indoor-sitting-duration values, comprising 77 values across 76 students. No student was excluded because of an invalid duration.

Missing continuous values were imputed using medians estimated within each training fold. Continuous variables were then standardised using parameters estimated from that same fold. Categorical encoding was fitted using the categories observed in the training fold. Although the preprocessing procedure allowed for imputation using the most frequent category within that fold, no categorical imputation was required. “Unknown” and “no homework” were retained as distinct response categories for homework duration. Categories encountered only in an evaluation school were represented by all-zero indicators for the corresponding predictor, without updating the fitted encoder.

Of the 11,445 source questionnaire records, 2,523 were from students in primary grades 1–3, outside the questionnaire’s specified scope, and 11 had identifiers that could not be reliably confirmed. Among the remaining 8,911 eligible records, 1,793 were later observations from students who already had an eligible record. Retaining the first eligible record for each student produced an analytical sample of 7,118 students. Eligibility was assessed before the first record was selected. Thus, 426 students with an earlier, ineligible record from a lower primary grade were included at their first eligible survey. The source records and the reasons for analytical inclusion or exclusion were retained.

Preprocessing parameters were re-estimated within every model-fitting subset and applied unchanged to the corresponding evaluation data. Student identity, school identity, survey date and monitoring round were not included as predictors. The final model was fitted using the full eligible cohort after the IECV procedure had been specified.

### Outcome definition

The outcome was defined by the questionnaire item: “Without wearing glasses, is the vision in one or both of your eyes below 5.0?” An affirmative response was classified as positive, indicating self-reported reduced uncorrected visual acuity in at least one eye; a negative response was classified as negative. The threshold of 5.0 uses Chinese logarithmic visual-acuity notation. The outcome was defined solely by the questionnaire response, without measured refraction or a spherical-equivalent criterion.

### Internal–external cross-validation and model development

In the outer IECV loop, each school was held out in turn, and model development used data from the other six schools. Each student therefore received a prediction from a model developed without data from that student or their school. Within each outer training set, an inner leave-one-school-out loop compared candidate model configurations and probability-calibration strategies. This design evaluated the complete model-development procedure across seven schools within a single multicentre dataset (13). The final model was developed using the full cohort and the same selection procedure. Its fitted values were kept separate from the predictions used to estimate IECV performance.

### Feature specification and model construction

The fixed set of 16 predictors was evaluated using nine configurations: four GBM configurations, two CatBoost configurations and three configurations of L2-penalised logistic regression (Supplementary Table S2). Selection was based on the lowest unweighted mean Brier score across the inner evaluation schools. Ties were resolved first by the higher mean within-school area under the receiver operating characteristic curve (AUC) and then by a fixed ordering of candidate configurations. An academic-year-only logistic-regression benchmark used the same outer school partitions, with calibration and threshold estimation performed within the training data. Comparisons of individual model families restricted inner selection to configurations from that family. Analyses were conducted in Python using scikit-learn, CatBoost, SciPy, statsmodels and SHAP. The use of generative AI to assist with analysis-code development is disclosed in the Generative AI statement. The random seed was set to 20260920.

Two probability-calibration strategies were considered: retaining the original model probabilities and applying logistic recalibration. Logistic recalibration modelled the outcome as a function of the logit of the predicted probability. Its slope was constrained to be nonnegative so that recalibration could not reverse the ordering of predictions. For each inner evaluation school, the recalibration function was fitted using an additional set of school-held-out predictions generated within the remaining inner training schools. Neither the base model nor the recalibration function was fitted using data from the inner evaluation school. Once a model configuration and calibration strategy had been selected, school-held-out predictions generated within the complete outer training set were used to fit the selected probability transformation and estimate the Youden-optimal threshold. When several thresholds maximised the Youden index, the lowest finite threshold was chosen. The resulting model, probability transformation and threshold were applied unchanged to the outer evaluation school. This procedure was repeated across all seven schools. Pooled outer predictions were used for evaluation, not threshold selection.

### Model evaluation and interpretability

Discrimination was assessed using AUC, and overall probabilistic prediction accuracy was assessed using the Brier score. Classification measures were calculated using the threshold estimated in the corresponding outer training set for each student. Confidence intervals for AUC and the Brier score were estimated using 2,000 bootstrap samples, with students resampled within schools. These intervals were conditional on the observed schools and the fixed predictions for students in held-out schools; they did not quantify performance uncertainty for a randomly selected new school.

Calibration was assessed using calibration-in-the-large, the calibration slope and the observed-to-expected ratio. Calibration-in-the-large was estimated with the prediction logit included as an offset, whereas the calibration slope was estimated together with an intercept. Confidence intervals for the calibration regression estimates were model-based 95% Wald intervals. Calibration was evaluated overall, by school and by educational stage. Calibration plots grouped predictions into probability deciles and displayed Wilson intervals for the observed outcome proportions.

Exploratory decision-curve net benefit was calculated as the true-positive proportion minus the false-positive proportion weighted by the threshold odds (14). Both proportions used the total sample size as their denominator. The reference outcome was the self-reported questionnaire response, so these calculations describe classification trade-offs under hypothetical decision thresholds. Their clinical interpretation requires evaluation against standardised vision measurements.

SHAP was used to explain predictions from the final model in 500 randomly selected students, with a background sample of 100 students (15). Contributions from encoded categories were aggregated to the original 16 predictors on the model log-odds scale, incorporating the selected recalibration strategy. The same primary-school student was used in both individual explanation plots. The complete model-selection procedure was repeated in three prespecified sensitivity analyses: restricting the sample to the 2021 survey round, excluding academic year and excluding vision-check history.

## Results

### Baseline characteristics and behavioural phenotypes

The analytical cohort comprised 7,118 students. Of these, 4,168 (58.6%) reported uncorrected visual acuity below 5.0 in at least one eye, and 2,950 (41.4%) did not. The cohort included 1,586 primary-school students, 2,150 junior-high students, 2,192 senior-high students and 1,190 vocational students. Each student contributed one eligible observation. Table 1 summarises demographic and behavioural characteristics by questionnaire outcome.

**Table 1:** Demographic and behavioural characteristics by self-reported visual-acuity outcome.

Variables	Total (*n* = 7,118)	Negative (*n* = 2,950)	Positive (*n* = 4,168)
Educational stage			
Primary school	1,586 (22.3)	985 (33.4)	601 (14.4)
Junior high school	2,150 (30.2)	860 (29.2)	1,290 (31.0)
Senior high school	2,192 (30.8)	617 (20.9)	1,575 (37.8)
Vocational school	1,190 (16.7)	488 (16.5)	702 (16.8)
Sex			
Male	3,546 (49.8)	1,612 (54.6)	1934 (46.4)
Female	3,572 (50.2)	1,338 (45.4)	2,234 (53.6)
Desk-chair adjustment			
Never/not adjustable	4,912 (69.0)	1754 (59.5)	3,158 (75.8)
Annually	661 (9.3)	352 (11.9)	309 (7.4)
Each semester	934 (13.1)	500 (16.9)	434 (10.4)
Every 2–3 months	611 (8.6)	344 (11.7)	267 (6.4)
Location during breaks			
Inside teaching building	5,069 (71.2)	1840 (62.4)	3,229 (77.5)
Outdoors	2049 (28.8)	1,110 (37.6)	939 (22.5)
Parental myopia			
Father only	1,105 (15.5)	423 (14.3)	682 (16.4)
Mother only	1,227 (17.2)	416 (14.1)	811 (19.5)
Both parents	958 (13.5)	301 (10.2)	657 (15.8)
Neither parent	3,828 (53.8)	1810 (61.4)	2018 (48.4)
Homework time per day			
<1 h	1808 (25.4)	880 (29.8)	928 (22.3)
1 to <2 h	2,763 (38.8)	1,138 (38.6)	1,625 (39.0)
2 to <3 h	1,426 (20.0)	506 (17.2)	920 (22.1)
≥3 h	745 (10.5)	247 (8.4)	498 (11.9)
Unknown	210 (3.0)	72 (2.4)	138 (3.3)
No homework	166 (2.3)	107 (3.6)	59 (1.4)
Tutoring attendance			
None	4,119 (57.9)	1,573 (53.3)	2,546 (61.1)
Reports a duration	2,835 (39.8)	1,302 (44.1)	1,533 (36.8)
Unknown duration	164 (2.3)	75 (2.5)	89 (2.1)
Reading distance >33 cm			
Never	521 (7.3)	228 (7.7)	293 (7.0)
Occasionally	2,923 (41.1)	999 (33.9)	1924 (46.2)
Often	2,118 (29.8)	888 (30.1)	1,230 (29.5)
Always	1,556 (21.9)	835 (28.3)	721 (17.3)
Parental posture reminders			
Never	683 (9.6)	318 (10.8)	365 (8.8)
Occasionally	2068 (29.1)	783 (26.5)	1,285 (30.8)
Often	2,397 (33.7)	953 (32.3)	1,444 (34.6)
Always	1970 (27.7)	896 (30.4)	1,074 (25.8)
Reading or screen use lying down			
Never	2,629 (36.9)	1,332 (45.2)	1,297 (31.1)
Occasionally	3,383 (47.5)	1,275 (43.2)	2,108 (50.6)
Often	907 (12.7)	276 (9.4)	631 (15.1)
Always	199 (2.8)	67 (2.3)	132 (3.2)
Evening lighting			
Desk and ceiling lamps	4,212 (59.2)	1739 (58.9)	2,473 (59.3)
Desk lamp only	830 (11.7)	358 (12.1)	472 (11.3)
Ceiling lamp only	1876 (26.4)	739 (25.1)	1,137 (27.3)
Other	200 (2.8)	114 (3.9)	86 (2.1)
Near-work break interval			
≤15 min	1,203 (16.9)	602 (20.4)	601 (14.4)
>15 to <30 min	1,615 (22.7)	730 (24.7)	885 (21.2)
0.5 to <1 h	2011 (28.3)	812 (27.5)	1,199 (28.8)
1 to <2 h	1,329 (18.7)	482 (16.3)	847 (20.3)
2 to <3 h	385 (5.4)	123 (4.2)	262 (6.3)
≥3 h	575 (8.1)	201 (6.8)	374 (9.0)

Values are n (column %). Sex is based on questionnaire responses. Selected descriptive variables that were not included in the 16-predictor model are retained to characterise the study population.

### Model development and comparative performance

Model and calibration selection were repeated within each outer training set. Table 2 presents IECV performance for the complete selection workflow, the individual model families and the academic-year-only benchmark. Across the seven held-out schools, the unweighted mean Brier score was 0.213 and the mean within-school AUC was 0.658. Applying the same development procedure to all 7,118 students selected a CatBoost model with 200 trees, a tree depth of 5 and a learning rate of 0.05, while retaining the original model probabilities. Supplementary Table S2 lists the candidate configurations, the choices made for each held-out school and the configuration of the final model.

**Table 2:** Discrimination and probabilistic prediction accuracy based on IECV predictions.

Model or procedure	AUC (95% CI)	Brier score
Selected workflow	0.706 (0.694–0.718)	0.212
GBM	0.698 (0.686–0.710)	0.214
CatBoost	0.704 (0.692–0.716)	0.213
Logistic regression	0.701 (0.689–0.714)	0.213
Academic year only	0.611 (0.598–0.624)	0.233

All procedures use the same outer school partitions. The selected workflow compares three model families; the model-family-specific procedures restrict inner selection to configurations within one family. AUC confidence intervals are conditional on the observed schools and fixed predictions.

### Discrimination across schools

The selected workflow achieved a pooled IECV AUC of 0.706 (95% CI 0.694–0.718; Figure 1), with AUCs within individual schools ranging from 0.625 to 0.701. The academic-year-only benchmark achieved an AUC of 0.611 (95% CI 0.598–0.624). The difference in AUC was 0.095 (conditional 95% CI 0.081–0.108), and the reduction in Brier score relative to the benchmark was 0.021 (95% CI 0.017–0.024). These comparisons quantify the additional predictive information provided by the full questionnaire beyond academic year.

**Figure 1:**
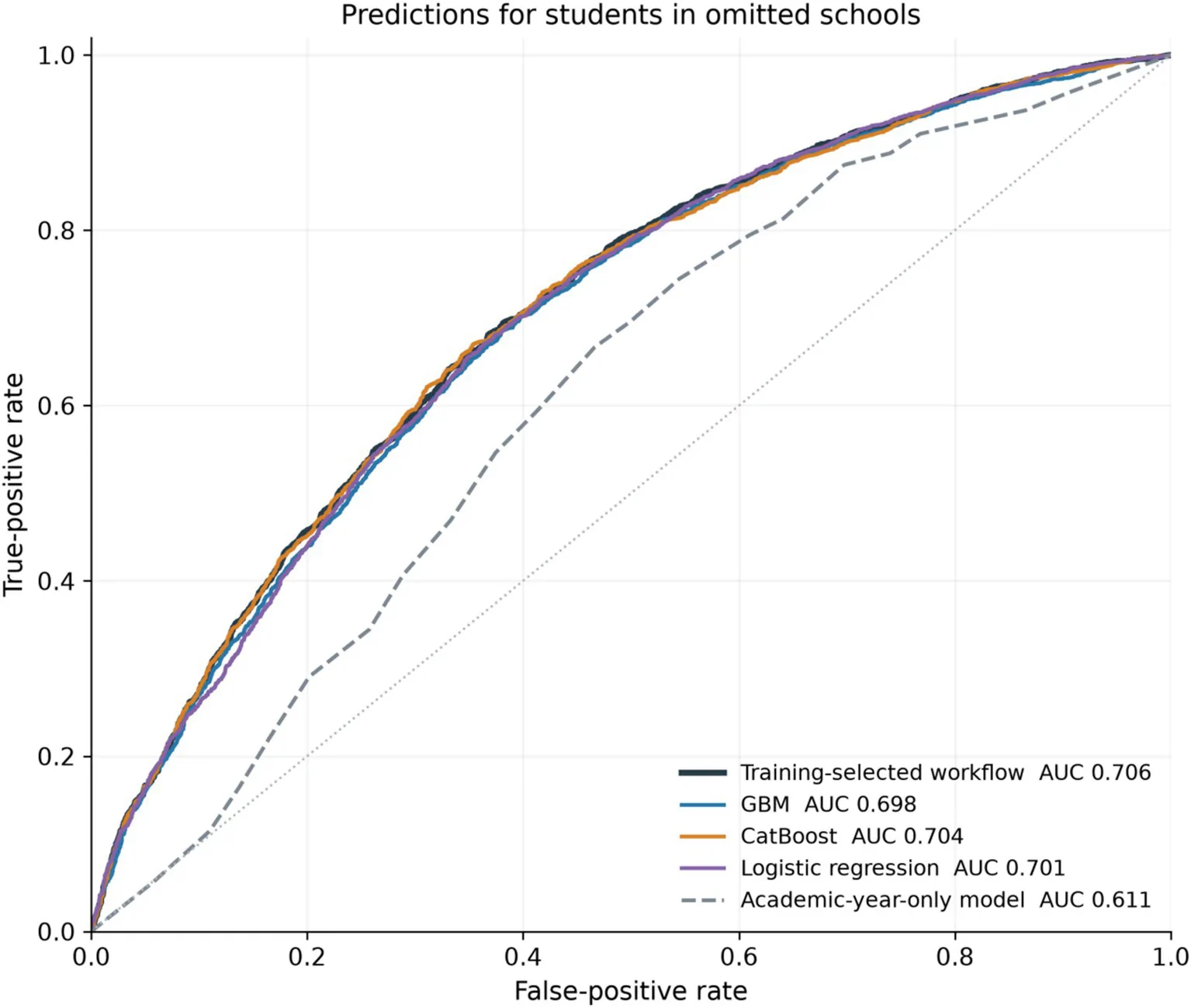
Receiver operating characteristic curves based on internal–external cross-validation (IECV) predictions. The selected workflow includes algorithm, hyperparameter and calibration selection within each outer training set. Model-family-specific procedures and the academic-year-only benchmark use the same held-out schools. The curves summarise performance across the seven outer IECV folds.

### Exploratory decision curve analysis

Decision curves based on IECV predictions compared the selected workflow with the academic-year-only benchmark and hypothetical assess-all and assess-none strategies (Figure 2). At an illustrative probability threshold of 0.40, net benefit was 0.336 per student for the selected workflow and 0.309 for the assess-all strategy. Net benefit was calculated against the self-reported questionnaire outcome, with false positives weighted by the threshold odds (14). The curves therefore describe classification trade-offs across analytical thresholds. Evaluation against standardised vision measurements and observed referral outcomes is needed to determine their clinical relevance. The classification thresholds used for performance assessment were estimated separately within the training schools.

**Figure 2:**
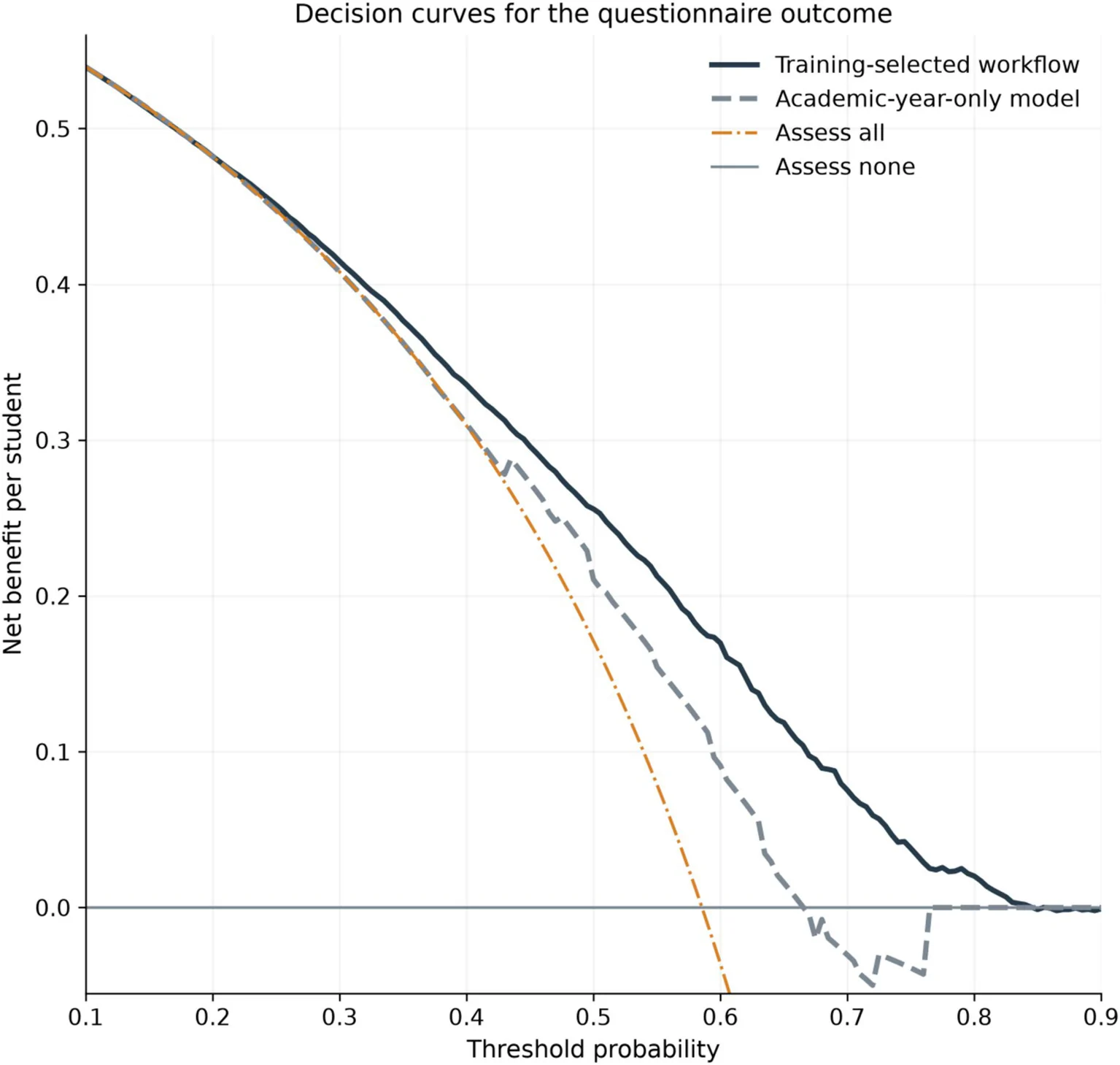
Exploratory decision curves based on IECV predictions for the self-reported visual-acuity outcome. Net benefit is shown for the selected workflow, the academic-year-only benchmark and hypothetical assess-all and assess-none strategies. The displayed thresholds are analytical values; classification thresholds were estimated within the training data. Net benefit represents classification trade-offs for the self-reported outcome. Its clinical relevance requires evaluation against standardised vision measurements and assessment of referral consequences.

### Model calibration

All seven outer training sets selected the original model probabilities rather than logistic recalibration. Consequently, the pooled IECV Brier score was unchanged at 0.212 before and after application of the selected calibration strategies. Calibration-in-the-large was −0.035, the calibration slope was 1.077 and the observed-to-expected ratio was 0.987 (Table 3). These estimates describe the agreement between predicted probabilities and observed questionnaire outcomes among students in held-out schools.

**Table 3:** Calibration of IECV predictions from the selected workflow, overall and by educational stage, with comparisons using the original probabilities and individual model families.

Setting/procedure	AUC	Brier	CITL (95% CI)	Slope (95% CI)	O/E	N
Selected workflow	0.706	0.212	−0.035 (−0.085–0.015)	1.077 (1.002–1.153)	0.987	7,118
Before probability conversion	0.706	0.212	−0.035 (−0.085–0.015)	1.077 (1.002–1.153)	0.987	7,118
Primary school	0.659	0.220	−0.171 (−0.278−0.064)	0.853 (0.691–1.015)	0.911	1,586
Junior high school	0.680	0.217	−0.026 (−0.115–0.063)	1.214 (1.040–1.388)	0.990	2,150
Senior high school	0.660	0.191	0.205 (0.108–0.301)	0.999 (0.827–1.171)	1.059	2,192
Vocational school	0.633	0.232	−0.268 (−0.388−0.149)	0.938 (0.703–1.172)	0.909	1,190
GBM	0.698	0.214	0.005 (−0.046–0.055)	0.940 (0.872–1.008)	1.002	7,118
CatBoost	0.704	0.213	0.039 (−0.011–0.090)	0.961 (0.893–1.029)	1.014	7,118
Logistic regression	0.701	0.213	−0.018 (−0.068–0.033)	0.960 (0.892–1.029)	0.994	7,118
Academic year only	0.611	0.233	−0.014 (−0.063–0.034)	0.863 (0.761–0.965)	0.995	7,118

CITL, calibration-in-the-large; O/E, observed-to-expected ratio. Ideal values are 0 for CITL and 1 for both the calibration slope and O/E. The calibration regressions assess the evaluation predictions without updating them.

Table 3 and Figure 3 present calibration results for the full cohort and all four educational stages. Predictions for each held-out school retained the probability transformation selected within its corresponding training schools. Calibration regressions and plots were used to assess these predictions without updating them.

**Figure 3:**
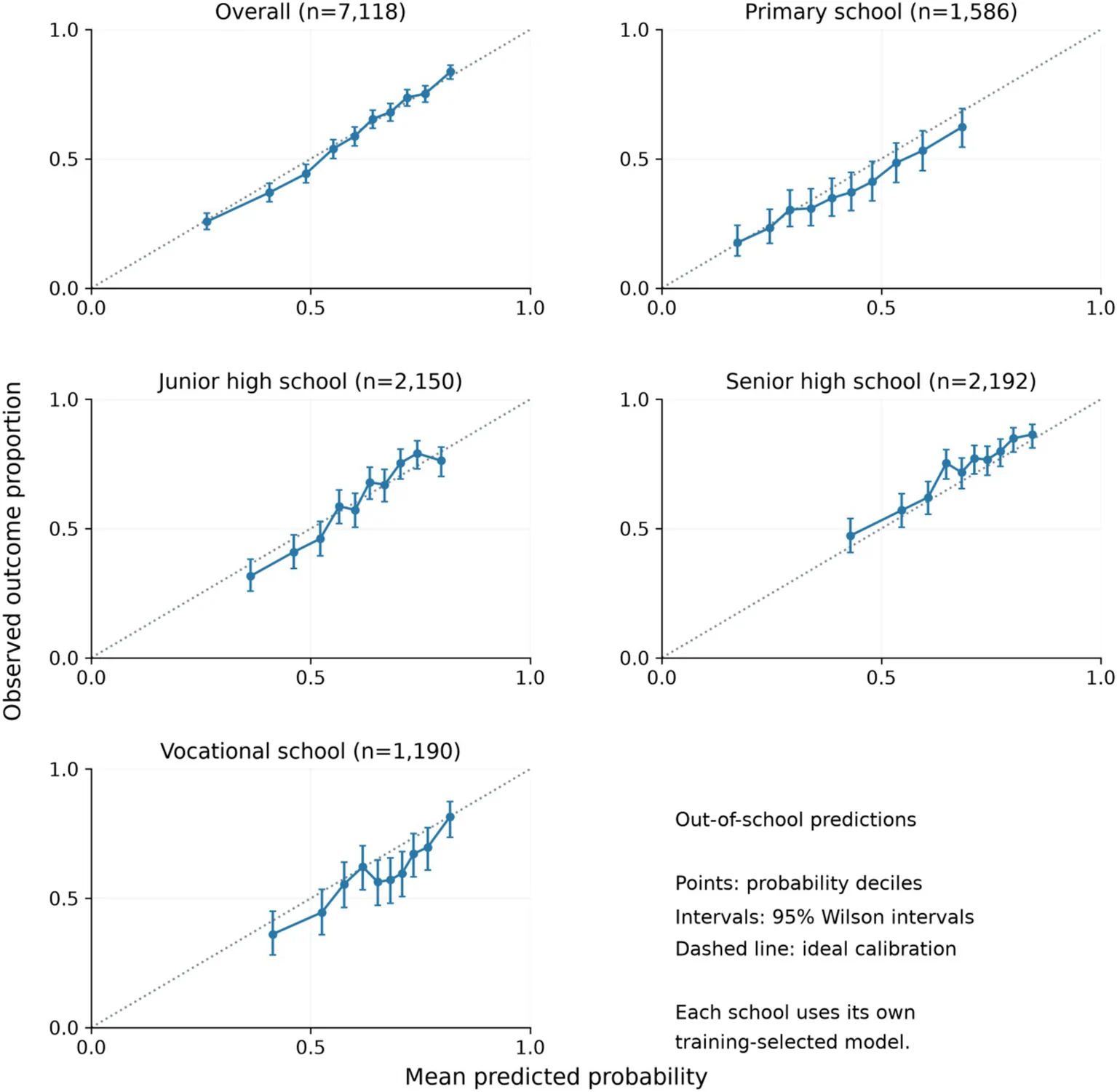
Calibration plots based on IECV predictions for the full cohort and each educational stage. Points show the mean predicted probability and observed outcome proportion within probability deciles. Error bars represent 95% Wilson intervals conditional on these groups. Predictions for each held-out school retain the probability transformation selected within its training schools. The diagonal represents ideal calibration.

### Classification thresholds and performance

The Youden thresholds estimated in the seven outer training sets ranged from 0.553 to 0.609. Applying each threshold to its corresponding held-out school yielded 3,044 true positives, 1,253 false positives, 1,697 true negatives and 1,124 false negatives relative to the questionnaire outcome. Pooled sensitivity was 0.730, specificity was 0.575, positive predictive value was 0.708, negative predictive value was 0.602, the F1 score was 0.719 and accuracy was 0.666. Table 4 presents performance at the fold-specific thresholds alongside descriptive results for a prespecified grid of common thresholds. The threshold estimated separately for the final model fitted to the full cohort was 0.600147. This threshold was not used to recalculate IECV performance.

**Table 4:** Classification performance based on IECV predictions.

Threshold	Sensitivity	Specificity	PPV	NPV	F1	Accuracy	Youden J
Fold-specific	0.730	0.575	0.708	0.602	0.719	0.666	0.306
0.20	0.994	0.036	0.593	0.809	0.743	0.597	0.030
0.30	0.974	0.125	0.611	0.771	0.751	0.622	0.099
0.40	0.929	0.247	0.635	0.710	0.754	0.646	0.175
0.50	0.847	0.421	0.674	0.660	0.751	0.670	0.268
0.60	0.681	0.632	0.723	0.584	0.702	0.661	0.313
0.70	0.398	0.837	0.775	0.496	0.526	0.580	0.235
0.80	0.105	0.975	0.857	0.435	0.186	0.465	0.080

Fold-specific thresholds were estimated within the training data; common fixed thresholds are shown for descriptive comparison. PPV, positive predictive value; NPV, negative predictive value. Youden J = sensitivity + specificity − 1.

At the training-derived thresholds, 4,297 of 7,118 students (60.4%) were classified as positive. This proportion, together with the specificity of 0.575, indicates that many students with a negative questionnaire response also received a positive model classification. The implications for school-health services require evaluation using standardised vision measurements, available examination capacity, attendance and the consequences of missed or unnecessary referrals.

### Stratified performance by educational stage and school

IECV AUCs were 0.659, 0.680, 0.660 and 0.633 among primary-school, junior-high, senior-high and vocational students, respectively. The corresponding proportions of positive questionnaire responses were 37.9%, 60.0%, 71.9% and 59.0%. Table 5 presents results by educational stage and individual school, distinguishing within-stage discrimination from the pooled estimate.

**Table 5:** IECV performance of the selected workflow by educational stage and individual school.

Stratum	N	Positive	AUC (95% CI)	Sensitivity	Specificity
Overall	7,118	4,168	0.706 (0.694–0.718)	0.730	0.575
Primary school	1,586	601	0.659 (0.632–0.688)	0.246	0.906
Junior high school	2,150	1,290	0.680 (0.657–0.702)	0.719	0.541
Senior high school	2,192	1,575	0.660 (0.636–0.685)	0.881	0.295
Vocational school	1,190	702	0.633 (0.602–0.666)	0.828	0.324
Primary school 1	752	246	0.633 (0.591–0.675)	0.073	0.976
Senior-high school 1	1,107	811	0.701 (0.667–0.735)	0.911	0.260
Primary school 2	834	355	0.659 (0.622–0.694)	0.366	0.831
Junior-high school 1	930	528	0.678 (0.644–0.714)	0.667	0.604
Junior-high school 2	1,220	762	0.676 (0.647–0.706)	0.755	0.485
Senior-high school 2	1,085	764	0.625 (0.587–0.661)	0.849	0.327
Vocational school	1,190	702	0.633 (0.602–0.666)	0.828	0.324

Classification uses the threshold estimated in each student’s corresponding outer training set. Stage-specific estimates pool predictions from the relevant schools; vocational education is represented by one school. AUC confidence intervals are conditional on the observed schools.

Brier scores were 0.220, 0.217, 0.191 and 0.232 among primary-school, junior-high, senior-high and vocational students, respectively. The corresponding calibration-in-the-large estimates were −0.171, −0.026, 0.205 and −0.268, and the calibration slopes were 0.853, 1.214, 0.999 and 0.938. Sensitivity and specificity at the training-derived thresholds are presented in Table 5. Sensitivity was 0.246 among primary-school students and 0.881 among senior-high students, whereas specificity was 0.906 and 0.295, respectively. These results demonstrate substantial variation in classification performance across educational stages. Primary, junior-high and senior-high education were each represented by two schools, whereas the vocational results were based on a single school.

### Sensitivity analyses and the contribution of academic year

Repeating the complete development procedure without academic year yielded an IECV AUC of 0.693 (95% CI 0.681–0.706); excluding vision-check history yielded an AUC of 0.701 (95% CI 0.689–0.714). In the prespecified analysis restricted to the first survey round, 3,134 eligible students each contributed one record, and the AUC was 0.684 (95% CI 0.665–0.701; Table 6). These analyses assessed the contributions of academic year and previous vision checks, as well as model performance within a single monitoring round.

**Table 6:** Benchmark and sensitivity analyses.

Analysis	N	Brier	AUC (95% CI)
Full specification	7,118	0.212	0.706 (0.694–0.718)
Academic year only	7,118	0.233	0.611 (0.598–0.624)
Without academic year	7,118	0.216	0.693 (0.681–0.706)
Without vision-check history	7,118	0.214	0.701 (0.689–0.714)
2021 survey round	3,134	0.221	0.684 (0.665–0.701)

Each row reports IECV performance after repeating the complete development procedure within the training data for the specified analysis. The 2021 survey was prespecified as the first survey round for the single-round analysis. Analyses excluding a predictor also repeated model and calibration selection.

### Global interpretability of behavioural predictors

SHAP analysis was used to interpret the final CatBoost model. Figure 4 ranks all 16 predictors by their mean absolute contribution to model log-odds. Academic year, parental myopia and desk-chair adjustment were the leading contributors. Figure 5 shows the contributions of vision-check history within categories of parental myopia. Figure 6 presents the distribution of contributions across 500 randomly selected students, with orange and blue indicating increases and decreases relative to the model reference value, respectively. Together, these plots show how demographic, familial and behavioural information contributes to the fitted predictions.

**Figure 4:**
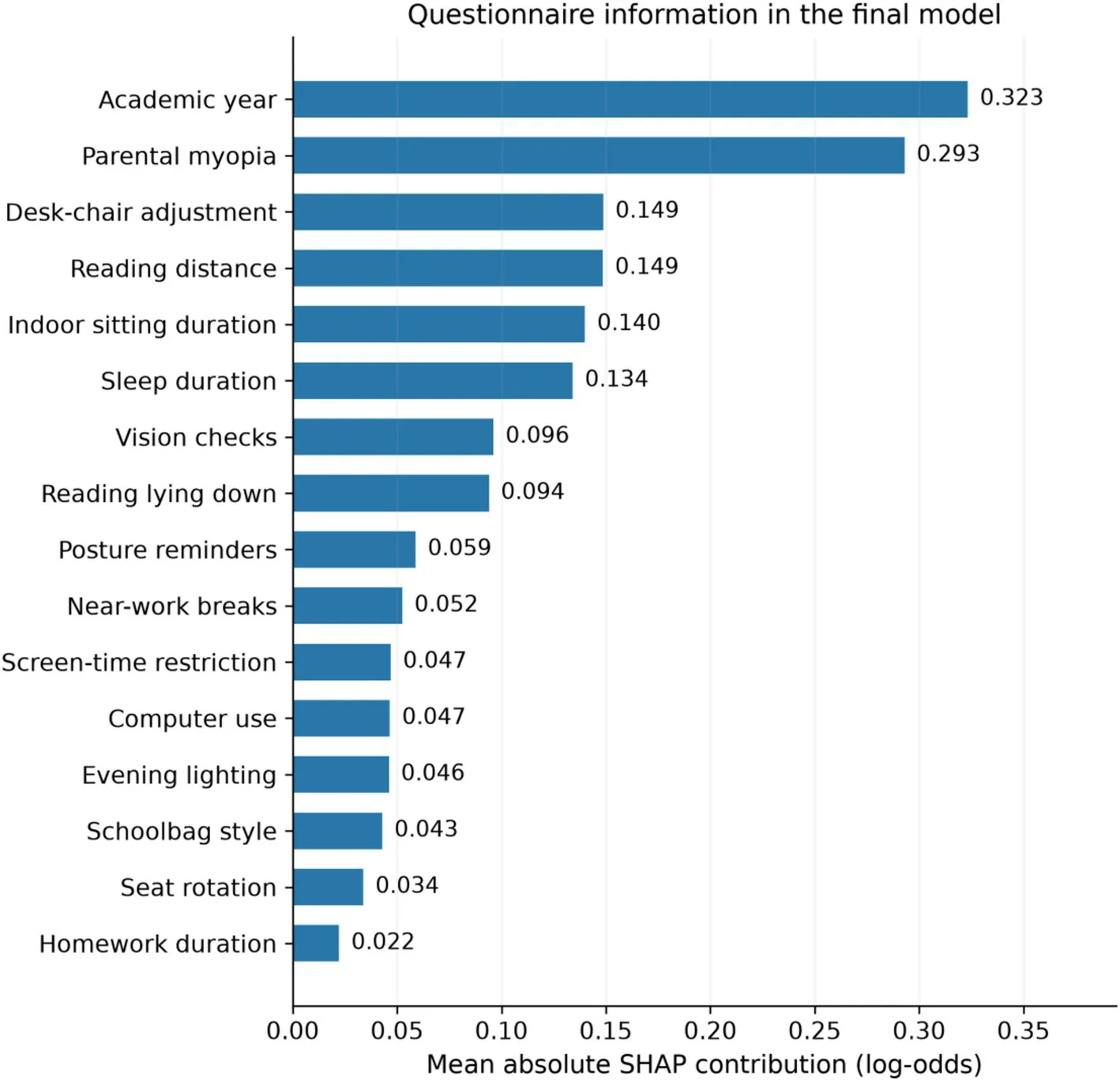
Global feature importance for the final CatBoost model in 500 randomly sampled students. Bars and numerical labels show mean absolute SHAP contributions to model log-odds. Contributions from encoded categories are summed within each of the 16 questionnaire predictors.

**Figure 5:**
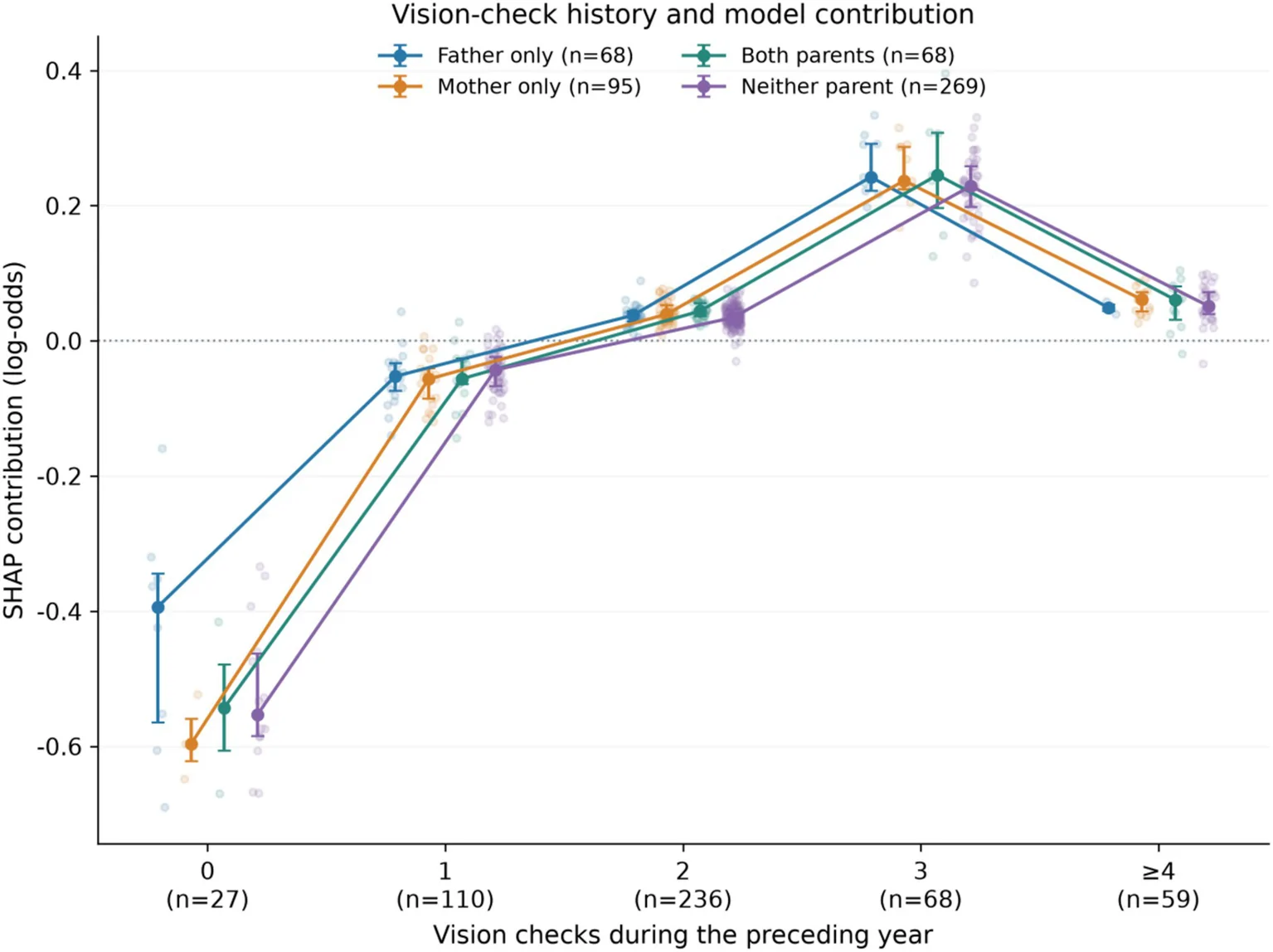
SHAP contributions of vision-check frequency in the 500-student explanation sample, stratified by parental-myopia category. Faint points show individual contributions, connected markers show medians, and whiskers show interquartile ranges. Counts below the frequency categories indicate the total number of students in each category; legend counts indicate the sizes of the parental-myopia groups. Frequency labels denote the number of vision checks. Parental-myopia categories are nominal and do not imply an ordering of severity.

**Figure 6:**
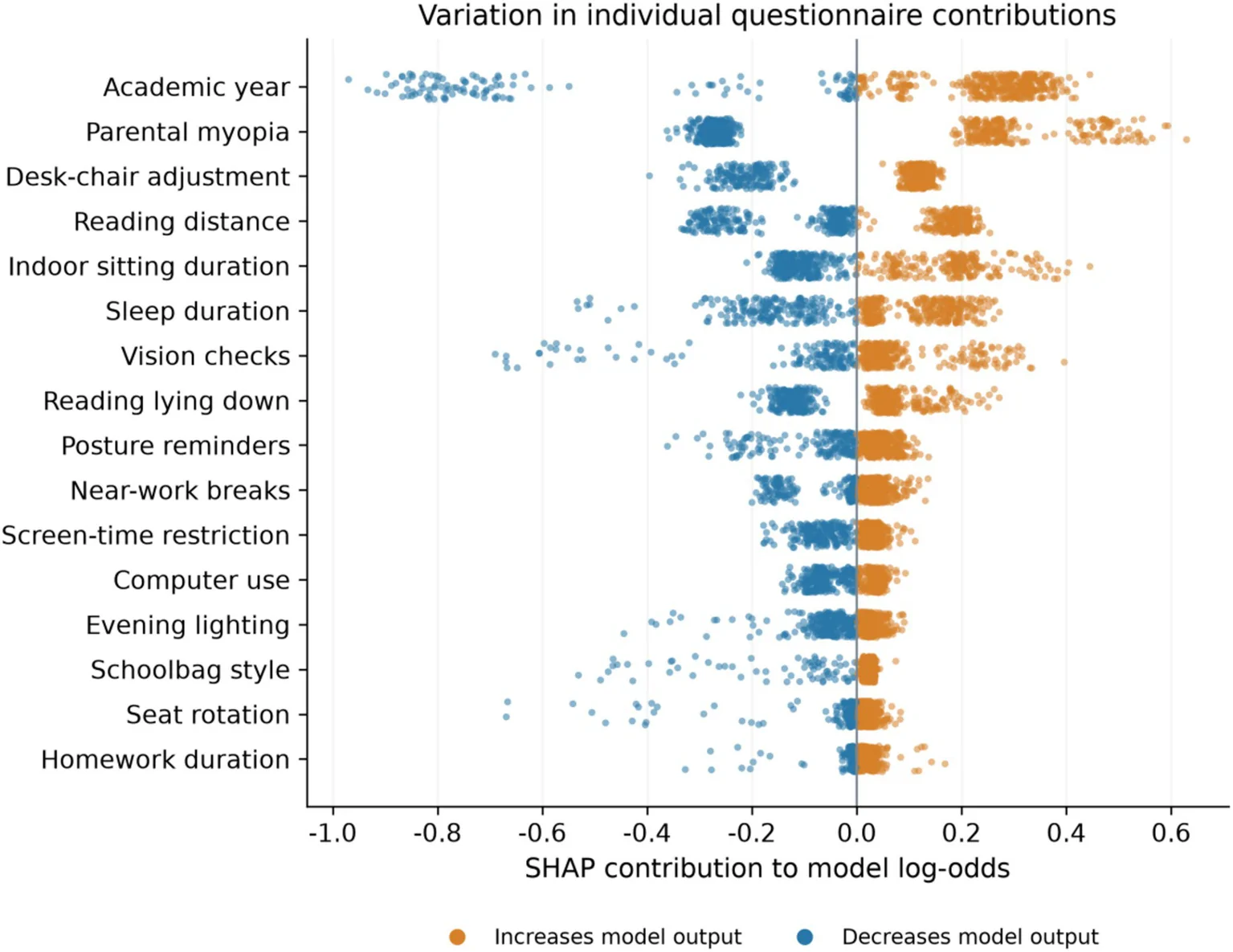
Distribution of SHAP contributions in 500 randomly sampled students. Predictors are ordered by mean absolute contribution. Horizontal position indicates the contribution to model log-odds; orange denotes a positive contribution and blue a negative contribution. Colour represents the direction of contribution, not the numerical coding of questionnaire responses.

### Individual probability profiles and visualisation

Figures 7, 8 present explanations for the same Grade 4 primary-school student selected from the SHAP sample. The predicted probability of a positive questionnaire response was 33.9%. The SHAP reference value was 0.336 on the model log-odds scale, equivalent to a probability of 58.3% after inverse-logit transformation. Adding the eight contributions with the largest absolute values and the sum of the remaining eight contributions to this reference value, then applying the inverse-logit transformation, reproduces the predicted probability. These plots illustrate how the student’s recorded responses contribute to the prediction.

**Figure 7:**
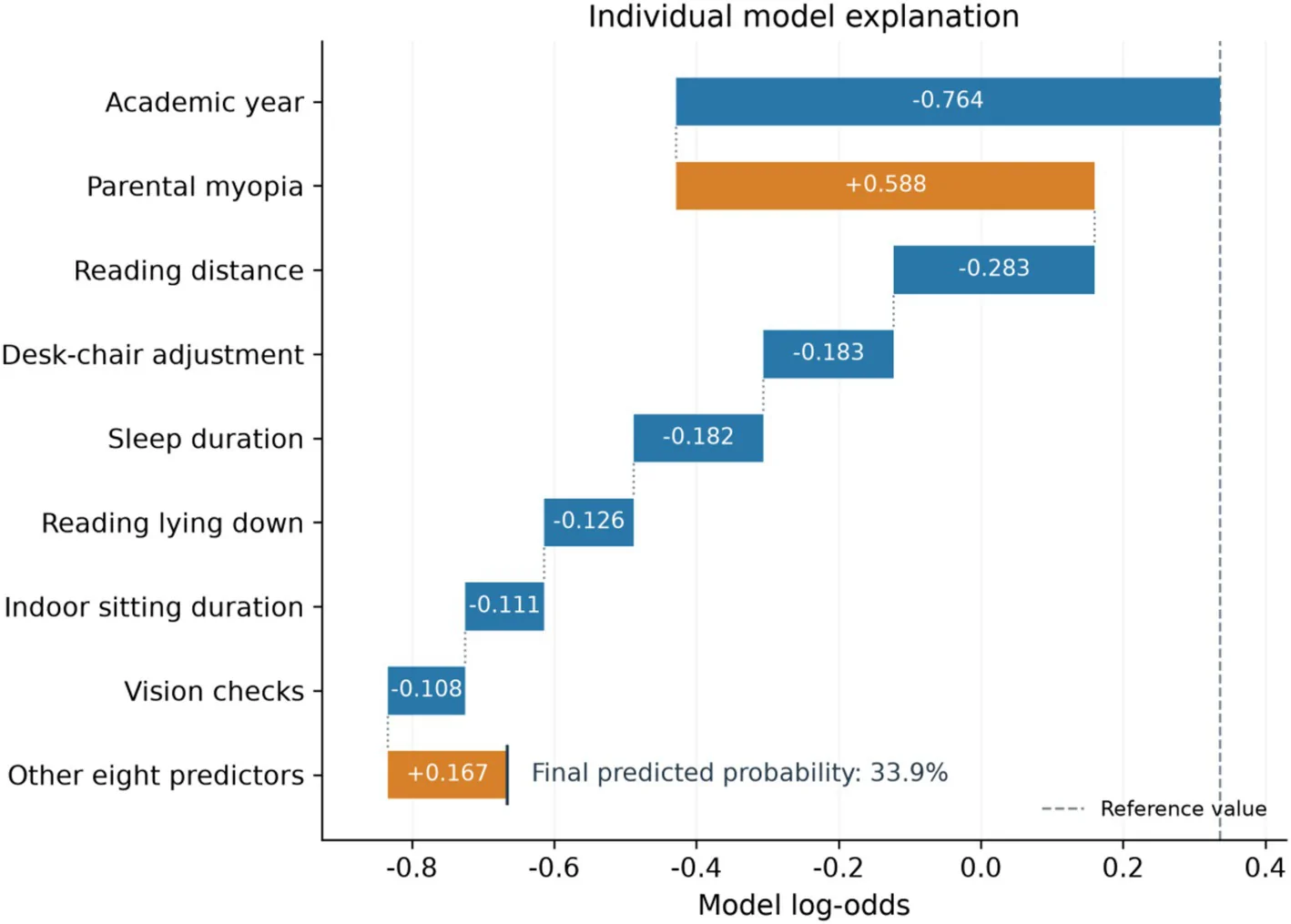
SHAP decomposition for a Grade 4 primary-school student who reported myopia in both parents. The predicted probability of a positive questionnaire response was 33.9%. The eight contributions with the largest absolute values and the combined contribution of the remaining eight predictors are shown. Orange and blue indicate contributions that increase and decrease model log-odds relative to the reference value, respectively.

**Figure 8:**
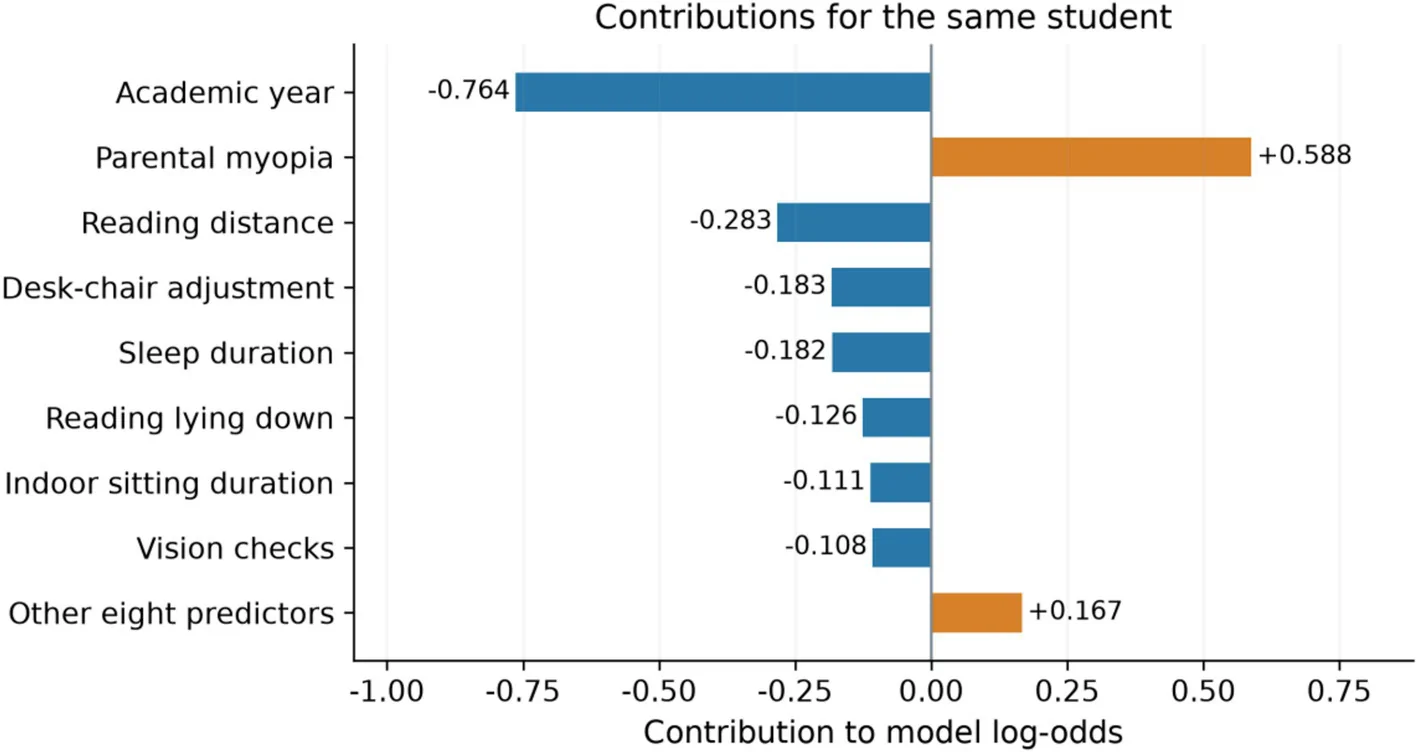
Signed SHAP contributions for the student and model shown in Figure 7. The eight contributions with the largest absolute values and the combined contribution of the remaining eight predictors are displayed. These values describe contributions to the fitted prediction rather than estimates of intervention effects.

The individual profile shows the contribution of each questionnaire predictor to the fitted prediction. This transparent presentation provides a basis for research on communicating questionnaire-derived information; its effects on understanding and health behaviour remain to be evaluated.

## Discussion

This study evaluated an interpretable questionnaire-based framework in 7,118 students from seven schools. IECV with nested school-grouped model development yielded an AUC of 0.706, compared with 0.611 for the academic-year-only benchmark. The full questionnaire thus provided additional predictive information about self-reported visual status beyond academic year alone. Overall discrimination was moderate, specificity was limited, and classification performance varied substantially across educational stages. The public-health contribution lies in characterising the information contained in routine school questionnaires and the variation in model performance that can inform the design of subsequent school eye-health studies.

### Interpretable questionnaire-based modelling

SHAP quantifies each recorded predictor’s contribution to the final model’s log-odds, providing an explanation alongside the estimated probability of a positive questionnaire response. This links the model output to the information on which it is based and makes individual predictions more transparent. Figure 9 summarises cohort construction, IECV with nested school-grouped development, and interpretation of the final model.

**Figure 9:**
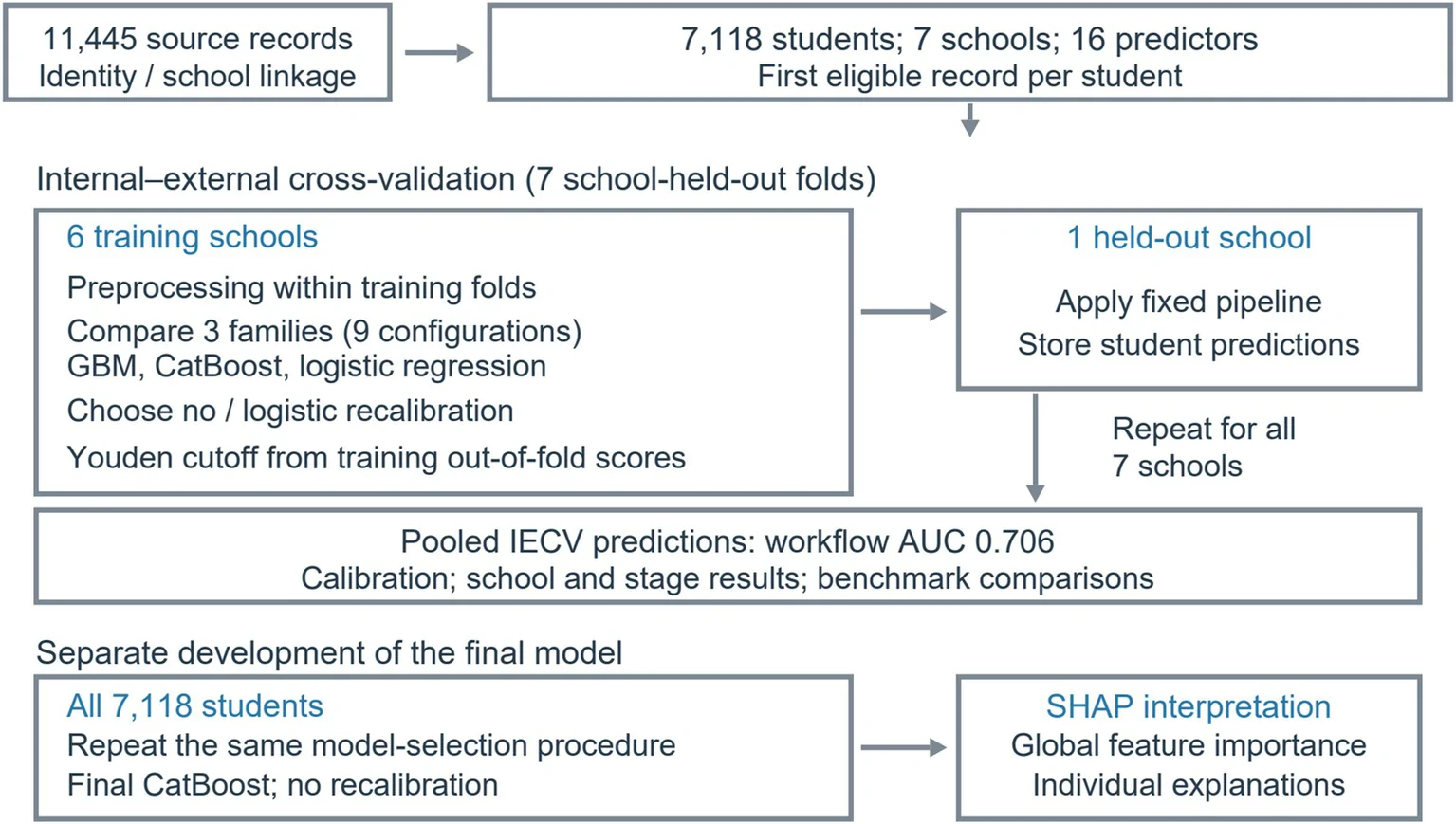
Questionnaire-based study framework using internal–external cross-validation (IECV). Sample construction retains the first eligible record for each student. In each of seven outer IECV folds, one school is held out and the other six are used for model development. Preprocessing, model selection, probability calibration and threshold estimation are confined to the training schools. Repeating this procedure yields predictions for students in all seven held-out schools. The final model is fitted separately to the full cohort using the same development procedure and is interpreted using SHAP.

Future school-health studies could link questionnaire profiles with routine vision screening, health education and follow-up records to evaluate their contribution to communication with families and the understanding of barriers to examination attendance. Such evaluations should assess performance against standardised vision measurements, together with service workload and coverage across educational stages. This would relate accessible questionnaire information to service organisation and access in settings with constrained eye-care resources. Any digital implementation would also require prospective evaluation of usability, feasibility and clinical consequences; a mobile-health application was not evaluated in this study.

### Interpretation and public-health implications

The global SHAP analysis highlights contributions from academic, familial and behavioural information. These questionnaire domains overlap with those examined in research on environmental characteristics (10–12), familial and behavioural factors (16), and sleep duration (17). Comparison with the academic-year-only benchmark demonstrates the incremental predictive information provided by the full questionnaire. The stage-specific calibration and classification results complement the pooled AUC by characterising substantial variation across school populations. This heterogeneity makes educational stage an important consideration when designing evaluations of questionnaire-based information within existing school-health services.

### Relationship to previous machine-learning studies and originality

Xi et al. used questionnaire data from 2,086 participants in eight schools to model parent-reported clinician-diagnosed myopia, using an individual-level training–testing split and focusing on LightGBM (18). The present study examined self-reported uncorrected visual acuity below 5.0 in at least one eye among 7,118 students. Its design combined one eligible record per student, IECV with nested school-level selection of algorithms and calibration strategies, comparison with an academic-year-only benchmark, and evaluation across four educational stages. The contribution is the systematic evaluation and interpretation of accessible school-health information across school settings using established statistical and machine-learning methods.

This study complements research on the external validation of myopia prediction models (19) and imaging-based approaches using optic-disc images (20) or retinal features (21). Differences in outcome definitions, populations and data requirements limit direct comparison of published AUCs. Reporting calibration, overall accuracy of predicted probabilities and stage-specific classification performance alongside discrimination provides a more complete account of questionnaire-based modelling.

### Limitations and future directions

The outcome reflects self-reported current visual status and is susceptible to recall and reporting error. Predictor information and model families had been explored in previous analyses of these data, so the IECV findings require confirmation in a new independent dataset. The seven schools were located in one district and included only one vocational school; the conditional confidence intervals do not capture the full uncertainty associated with performance in new schools. The cohort contains each student’s first eligible survey record and excludes lower primary grades. SHAP contributions describe the fitted model, while reported habits and previous vision checks may partly reflect existing visual concerns. Prospective studies should compare the model with the self-report question alone and routine vision screening, using standardised visual assessments and school-health service outcomes to establish its added value.

## Conclusion

Under IECV, an interpretable questionnaire-based framework showed moderate discrimination for self-reported reduced uncorrected visual acuity in school students. The full questionnaire provided predictive information beyond academic year alone, while stage-specific analyses revealed substantial variation in classification performance. These findings support further investigation of routine questionnaires as an accessible source of information for school eye-health research. Evaluation against standardised visual assessments is needed to determine the framework’s potential supplementary role within existing school-health services.

## Data Availability

The dataset analysed in this study contains sensitive personal information about students and is not publicly available. Relevant data may be obtained from the corresponding author upon reasonable request, subject to formal ethical approval. The core code for model training and SHAP analysis is available from the corresponding author upon reasonable request.
