# Supplementary material for: Interpretable machine learning for self-reported reduced uncorrected visual acuity in school students
Source: Front Public Health. 2026 Oct 26;14:1872000. doi: 10.3389/fpubh.2026.1872000 (PMC13745713; doi:10.3389/fpubh.2026.1872000)
Supplement: Supplementary file 1 [file Table_1.pdf]

Supplementary Table S1. Definitions and representation of the 16 predictors. Response categories are represented by separate indicators; academic year and total daily durations are continuous variables. The analytical cohort includes students in primary grades 4–6. Predictor names are consistent across the text, tables and figures.

Predictor	Representation	Response categories and handling
Academic year	Continuous	Primary grades 4–6 correspond to academic years 4–6; junior-high grades 1–3 to years 7–9; senior-high and vocational grades 1–3 to years 10–12.
Classroom seat-rotation frequency	Indicators	Never or only selected pupils; once per semester; monthly; every two weeks; weekly.
Desk-chair height adjustment for student height	Indicators	Never or not adjustable; once per school year; once per semester; every two to three months.
After-school homework and reading-writing duration	Indicators	Mean daily after-school homework, reading and writing in the preceding week: <1, 1 to <2, 2 to <3, or ≥3 hours; unknown; no homework. Unknown and no homework are separate categories.
Parental limits on television, computer and video-game time	Indicators	Whether parents limit television, computer or video-game time: yes or no.

Predictor	Representation	Response categories and handling
Frequency of eye-to-book distance above 33 cm	Indicators	Frequency of keeping the eyes more than 33 cm from the book when reading/writing: never, occasionally, often or always.
Parental reminders about reading-writing posture	Indicators	Frequency of parental reminders about reading/writing posture: never, occasionally, often or always.
Daily computer-use duration	Indicators	Usual daily computer use in the preceding week: none, <1, 1 to <2, 2 to <3, 3 to <4, or ≥4 hours.
Reading or screen use while lying or prone	Indicators	Frequency of reading or viewing screens while lying or prone: never, occasionally, often or always.
Lighting for reading and writing at home after dark	Indicators	Desk and ceiling lamps together; desk lamp only; ceiling lamp only; other.
Interval between breaks during near work	Indicators	Continuous near work before a break: ≤15 minutes; >15 to <30 minutes; 0.5 to <1, 1 to <2, 2 to <3, or ≥3 hours.

Predictor	Representation	Response categories and handling
Parental myopia status	Indicators	Father only, mother only, both parents, or neither parent reported to have myopia; categories have no severity ordering.
Vision checks in the preceding year	Indicators	Number of vision checks in the preceding year: 0, 1, 2, 3, or ≥4.
Habitual schoolbag-carrying style	Indicators	Two-strap schoolbag carried in front, on the back, or on one side; single-strap bag; wheeled bag.
Daily sleep duration	Continuous	Total daily sleep hours = recorded hours + minutes/60. Hour component 0–24, minute component 0–59 and total ≤24; otherwise missing, imputed within each fitting fold.
Daily indoor sitting duration	Continuous	Total daily indoor sitting hours in the preceding week = recorded hours + minutes/60; the same validity checks and training-fold imputation apply.

Supplementary Table S2. Candidate configurations and selections made within the training data. For each candidate configuration, the table reports the lower of the two mean Brier scores obtained with the original probabilities or logistic recalibration. Scores were averaged across inner evaluation schools during development using the full cohort. School-specific rows show the configuration selected when the corresponding school was held out. The final row shows the model developed

separately using the full cohort. The last column reports a Brier score for candidate configurations and a classification threshold for fitted models.

**Candidate / fitted model**	**Specification**	**Brier / cutoff**
Logistic regression	L2 penalty; C=0.1	0.213
Logistic regression	L2 penalty; C=1	0.214
Logistic regression	L2 penalty; C=10	0.214
GBM	100 trees; depth 1; learning rate 0.05; minimum leaf 30	0.220
GBM	100 trees; depth 2; learning rate 0.05; minimum leaf 30	0.216
GBM	100 trees; depth 3; learning rate 0.05; minimum leaf 30	0.215
GBM	200 trees; depth 3; learning rate 0.05; minimum leaf 30	0.215
CatBoost	200 trees; depth 3; learning rate 0.05	0.213
CatBoost	200 trees; depth 5; learning rate 0.05	0.212
Outer training choices	Model and probability transformation	Cutoff
Primary school 1	Logistic regression; L2 penalty; C=0.1; original probabilities	0.609315
Senior-high school 1	CatBoost; 200 trees; depth 3; learning rate 0.05; original probabilities	0.552883

Candidate / fitted model	Specification	Brier / cutoff
Primary school 2	Logistic regression; L2 penalty; C=1; original probabilities	0.591142
Junior-high school 1	CatBoost; 200 trees; depth 3; learning rate 0.05; original probabilities	0.579441
Junior-high school 2	CatBoost; 200 trees; depth 3; learning rate 0.05; original probabilities	0.579109
Senior-high school 2	CatBoost; 200 trees; depth 5; learning rate 0.05; original probabilities	0.560664
Vocational school	CatBoost; 200 trees; depth 3; learning rate 0.05; original probabilities	0.568617
Final full-cohort model	CatBoost; 200 trees; depth 5; learning rate 0.05; identity mapping; $\text{logit}(p^*) = 0.000000 + 1.000000 \times \text{logit}(p)$	0.600147
